# Developing nurse and midwife centred rostering principles using co-design: a mixed-methods study

**DOI:** 10.1186/s12912-024-02522-7

**Published:** 2024-12-20

**Authors:** Sara Holton, Bodil Rasmussen, Karrie Long, Madison Bellizia, Jac C. Mathieson, Shane Crowe, Douglas Mill, Harry Pasion, Claire Rankin, Maree Woodhouse, Meaghan Douglas, Nadine Glanville, Kylie Baker, Kethly Fallon, Megan Hoffmann, Nicole Sliwa, Denise Heinjus, Lisa Fitzpatrick, Paul Gilbert

**Affiliations:** 1 School of Nursing and Midwifery, Deakin University, 1 Gheringhap Street, Geelong, Vic 3220 Australia; 2 Centre for Quality and Patient Safety Research – Western Health Partnership, Deakin University, 1 Gheringhap Street, Geelong, Vic 3220 Australia; 3 Clinical and Professional Leadership, Safer Care Victoria, 50 Lonsdale Street, 3000 Melbourne, Vic Australia; 4 Nursing and Midwifery, Western Health, PO Box 294, St Albans, Vic 3021 Australia; 5 Nursing and Midwifery, Echuca Regional Health, 226 Service Street, Echuca, Vic 3564 Australia; 6 Nursing Services, First Nations Health & Residential Aged Care, The Royal Melbourne Hospital, 300 Grattan Street, Parkville, Vic 3050 Australia; 7 Australian Nursing and Midwifery Federation (Victorian Branch), A’Beckett Street, PO Box 12600, Melbourne, Vic 8006 Australia

**Keywords:** Nurses, Midwifery, Health services, Hospitals, Occupational health, Fatigue, Shift work, Rostering

## Abstract

**Background:**

Current nursing and midwifery rosters are based on guidelines which may no longer adequately meet the needs of health services or staff and often result in decreased job satisfaction, poor health and wellbeing, and high turnover. Little is known about the rostering needs and preferences of contemporary nurses and midwives in Australia. The aim of this study was to identify the rostering concerns, needs and preferences of nurses and midwives, and co-design acceptable, equitable and feasible rostering principles.

**Methods:**

A mixed-methods design using a co-design approach with three components: survey, discussion groups, and co-design workshops. Nurses and midwives employed at three public health services in Victoria, Australia were invited to participate. The quantitative (survey) data were analysed using descriptive statistics and the qualitative (discussion groups and co-design workshops) data using thematic analysis.

**Results:**

Surveys were completed by 715 nurses and midwives including unit (*n* = 14) and roster (*n* = 13) managers. Nurses and midwives (*n* = 688) were mostly satisfied with their roster (mean satisfaction score = 57.4). Many had responsibilities or commitments which impacted their roster availability (*n* = 406, 61.6%) and over half had taken personal leave due to roster-related fatigue (*n* = 335, 59.1%) or unmet roster requests (*n* = 310, 54.7%). Midwives reported significantly less satisfaction (*p* < 0.001) and more challenges with current roster practices than nurses. Roster and unit managers described spending considerable time preparing and reworking rosters. Thirty-nine nurses and midwives participated in a focus group and outlined concerns about the fairness and equity of current roster practices, and the adverse impact on their health, work, and personal lives. Ninety-one nurses and midwives participated in a co-design workshop and identified a need for roster practices and guidelines which ensure flexibility, fairness and equity, and fatigue management.

**Conclusions:**

Although nurses and midwives were mostly satisfied with their rosters, they often experienced frustrations and challenges with current roster guidelines and practices as well as adverse effects on their health and work and personal lives. Nurses and midwives identified a preference for fair and equitable rosters which provide flexibility and enable them to manage their other commitments and responsibilities, reduce roster-related fatigue, and provide high quality patient care.

**Supplementary Information:**

The online version contains supplementary material available at 10.1186/s12912-024-02522-7.

## Background

The work of nurses and midwives is emotionally demanding, and they often experience high levels of occupational stress because of long work hours, heavy workloads, and irregular schedules [1, 2]. Rostering (scheduling) is an important process to enable efficient, effective and safe delivery of health care [3]. However, shift work is associated with several adverse health outcomes for nurses and midwives including burnout [4–7], fatigue [8, 9], musculoskeletal disorders [10], obesity [11, 12], higher rates of smoking [12], headaches [13], social isolation [13], mental distress [14] and sleep disorders [15–20]. Night shifts, early morning shifts, ‘quick returns’ and requests to work on scheduled days off are known to cause significant sleep loss and contribute to nurse fatigue [12, 15].

Nurses’ and midwives’ quality of professional life is also significantly affected by shift duration [21], time [22] and frequency [8]. Nurses and midwives working shifts also report lower levels of job satisfaction [14, 23] and job satisfaction is lower for those working fixed night shifts [24] and longer shift lengths [25]. An Australian study found nurses working rotating shifts experienced more psychological distress than those working a fixed roster [26].

Nurses’ and midwives’ work schedules have also been found to affect patient care. A South Korean study found that longer work hours were associated with missed nursing care [27]. Fatigue from working shift work has been shown to contribute to reduced productivity and patient safety and increased medical errors and workplace-related incidents [28].

Although the number of nurses and midwives employed and registered in the state of Victoria has increased recently [29–31], Australia’s health services are experiencing nursing and midwifery workforce shortages due to the reduced migration of skilled nurses and midwives, an increase in the number of nurses and midwives who intend to leave or have left the profession, and a great demand for nurses and midwives due to an increased number of patients [32]. Nurses and midwives have identified the need for improved working conditions [33] including the ability to better manage their paid work and personal lives and ‘avoid’ shift work [32].

Current nursing and midwifery rosters are based on guidelines which may no longer adequately meet the needs of nurses and midwives or health services. In Australia, legislation [34] and an industrial agreement [35] provide guidance for employers (e.g. health services) and nurses/midwives about rostering practices. These documents include principles and recommendations about individual flexible working arrangements, change of working hours and rosters, breaks, nurse/midwife (including skill mix): patient ratios, and rosters including fixed rosters and supplementary rosters. Health services also often have local policies and procedures which provide hospital wide or ward/unit based guidance and instruction around rostering practices in their setting.

There is evidence to suggest that more flexible working patterns and shift scheduling systems can improve nurses’ and midwives’ work-life balance, health and wellbeing, job satisfaction and retention [21, 36–41]. Yet little is known about the specific rostering concerns, needs and preferences of contemporary nurses and midwives especially those working in Australia. The aims of this study were to: (1) explore nurses’ and midwives’ experiences, perceptions of and satisfaction with current rostering guidelines and practices; (2) identify their rostering preferences; and then based on the findings, (3) co-design new acceptable and feasible rostering guidelines (which meet legislative requirements).

## Methods

### Study design

An exploratory sequential mixed-methods study using a co-design approach was conducted.

The study included three components:

1. Survey.
2. Focus groups.
3. Co-design workshops.

### Sample and study setting

The public hospital system in Australia provides free or low-cost care funded by the government. Nurses and midwives were recruited from three public health services in Victoria, Australia. The participating health services included two large tertiary health services in metropolitan Melbourne (Royal Melbourne Hospital (RMH) and Western Health (WH)) and one from regional Victoria (Echuca Regional Health (ERH)). All services provide acute, subacute, specialist and community health care and employed at the start of the study approximately 9,500 EFT nurses and midwives. A total of 14 wards/units were identified at the three study health services to participate in the study including three from health service #1 (ERH), four from study health service #2 (RMH) and seven at study health service #3 (WH).

All nurses and midwives employed at the participating health services who working in one of the identified wards were eligible to participate in the study.

### Procedure and data collection

#### Component 1 (survey)

Nurses and midwives (*n* = 726) working in the selected wards at the three study health services were invited to complete an anonymous online study-specific survey, hosted on REDCap, in February 2023. Separate surveys were conducted for the unit (NUMs/MUMs) and roster managers of these wards (*n* = 27). The surveys assessed respondents’ sociodemographic characteristics, perceptions, experiences and level of satisfaction with current roster guidelines and practices at their health service (Aim 1). The surveys included mostly fixed-response questions; however, space was provided at the end of the survey for respondents to make free text comments. The unit and roster manager surveys also included questions about the preparation of rosters, rostering needs and arrangements, and roster communications (Aim 1) (copies of the surveys are available in the Supplementary Material).

#### Component 2 (focus group)

Nurses and midwives and unit/roster managers from the selected wards at each study health service were invited to participate in a focus group discussion. These were conducted in ‘double-staffing time’ on different days of the week at the health services by members of the research team using a discussion guide and were audio-recorded and professionally transcribed for analysis. The discussion groups sought more in-depth data about nurses’ and midwives’ perceptions of, and satisfaction with, current roster guidelines and practices (Aim 1); and identified nurses’ and midwives’ rostering preferences (Aim 2). The focus groups with unit and roster managers sought further insights about the factors which inform and influence their roster decision-making and practices (the discussion guides are available in the Supplementary Material). The focus groups were held on MS Teams in May 2023.

#### Component 3 (co-design workshops)

All nurses and midwives working in the selected wards were invited to participate in a co-design workshop. A workshop was held at each study health service and at study health service 3 (WH) separate workshops were conducted for nurses and midwives. The workshops were conducted between July – August 2023 and hosted on the online platform WhatsApp. Nurses and midwives were invited to join the co-design workshop (WhatsApp ‘private’ group) via link (url) or QR code provided by the research team. The moderator outlined at the beginning of each workshop guidelines about participation such as respecting the privacy of other group members and not disclosing or sharing comments posted as part of the group discussion with those outside group. The moderator read participants’ responses daily and asked clarifying questions as appropriate.

Data from the survey and focus groups were used by the research team to draft roster guidelines and principles. The co-design workshops discussed, evaluated and refined these proposed and principles guidelines in terms of their comprehensibility, salience, and acceptability for nurses and midwives. A discussion guide (Supplementary Material) was used to prompt and initiate discussion about the proposed guidelines and whether changes were required before they could be used at the participating health services (Aim 3).

In order to develop a summary description of the discussion group (component 2) and co-design workshop (component 3) members, demographic data were sought in a brief survey outside of the discussion. The survey elicited sociodemographic and employment details about the participants including country of birth, number of years practicing as a nurse/midwife, and number of years employed at the study health service (the survey is available in the Supplementary Material).

### Data analysis

The quantitative and qualitative data were analysed separately. The results were then considered together to address the study’s objectives, and overall conclusions drawn.

#### Component 1 – survey (quantitative data)

Descriptive statistics were used to summarise and describe the survey data. Chi-square or Mann-Whitney U tests were used to test for significant differences between sub-groups (e.g. nurses vs. midwives). Quantitative data analysis was conducted using IBM SPSS Statistics version 25.

#### Components 2 and 3 – focus groups and co-design workshops (qualitative data)

The focus group and co-design workshop transcripts were de-identified, coded and analysed using thematic analysis techniques commonly practised in qualitative research [42]. This consisted of six phases. Phases 1 and 2: Transcripts were repeatedly read and reread, and coded. Phases 3–5: Codes were grouped into meaningful categories that described how participants talked about the topics, including contradictions and exceptions. Themes were created, named and defined in order to explain and interpret the content. Examples of the identified themes were selected in the final phase (phase 6) and related back to the research objective. The analysis was conducted by members of the research team and interpretations were discussed within the research team until consensus was reached. Participant quotes have been included to highlight the findings.

### Ethics approval

"The research was performed in accordance with the Declaration of Helsinki. Ethics approval for the project was received from the Bendigo Health Human Research Ethics Committee (LNR/96762/BH-2023-364908; 17 April 2023), the Royal Melbourne Hospital Human Research Ethics Committee (QA2023044, 27 April 2023) and the Western Health Low Risk Ethics Panel (QA2023.17_ 95075; 1 May 2023). Completion of the survey was taken as implied consent to participate in this component of the study which was approved by all ethics committees. Completion and signing of the Participant Information and Consent Form or verbal recorded consent was taken as informed voluntary consent to participate in the focus group and co-design workshop components of the study.

## Results

### Component 1 (survey)

#### Respondents

688 completed surveys were received from nurses and midwives (a response rate of 94.8%), 13 from roster managers (100% response rate) and 14 from unit managers (100% response rate) employed at the study health services.

#### Nurse and midwife survey

##### Respondents

Most respondents (*n* = 514, 74.7%) were registered nurses (RNs) or registered midwives (RMs) who mostly worked part time in medical wards or midwifery and on average worked about 54 h a fortnight. More than half were aged 35 years or less (*n* = 415, 60.4%), had worked at their current health service for five years or less (*n* = 431, 62.6%) and had worked as nurse/midwife for more than six years (*n* = 434, 63.1%) (Supplementary Material 1: Nurse and midwife survey data - Table 1).

##### Roster arrangements

Almost a third of the respondents (*n* = 189, 28.7%) had informal flexible work arrangements in place and just over one in ten had a formal flexible arrangement (*n* = 73, 11.1%) (Supplementary Material 1: Nurse and midwife survey data - Table 2).

Respondents stated that they mainly requested flexible work arrangements to manage their family responsibilities and for health reasons.

I have a 12 month FWA [flexible work arrangement] since returning to work after maternity leave. I have 2 set shifts a fortnight to help accommodate childcare. (Nurse and midwife survey respondent)

##### Respondents’ other responsibilities and commitments

More than half of the respondents (*n* = 406, 61.6%) stated that they had responsibilities or commitments which impacted their roster availability. These were mostly childcare or other care responsibilities, but respondents also reported that sporting commitments, religious, cultural and community activities and wanting a work life balance affected their roster availability (Supplementary Material 1: Nurse and midwife survey data - Table 3).

Everyone has different commitments outside of work, self selecting shifts, within reason would help and flexible work arrangements for those with children or who are a carer. (Nurse and midwife survey respondent)

SUPPORTING UNI students, so many workplaces just say “it’s a 7 day rotating roster” it’s a pain to get time off for university. (Nurse and midwife survey respondent)

##### Roster guidelines and request system

Only about a third of the respondents (*n* = 202, 30.6%) stated that their ward or unit had roster guidelines but most (*n* = 608, 98.1%) said there was a rostering request system in their ward or unit (Supplementary Material 1: Nurse and midwife survey data - Table 4).

##### Roster requests

Most respondents indicated that on average per fortnight they were submitting up to five roster requests per fortnight (*n* = 589, 91.9%) and these requests were usually or always supported (*n* = 382, 59.5%) (Supplementary Material 1: Nurse and midwife survey data - Table 5). Nevertheless, many respondents wrote free text comments about the difficulties they experienced getting their requests approved.

Ability to request is good however I rarely get these requests. (Nurse and midwife survey respondent)

I work 2 × 12-hour shift as maternity access and often put in requests for 5 options in the fortnight - rarely do I get rostered one of the 5 requested. (Nurse and midwife survey respondent)

##### Roster satisfaction

The survey respondents were asked to rate their level of satisfaction with their roster on a 100-point scale which ranged from not satisfied (0) to satisfied (100). The mean score was 57.4 indicating that respondents were mostly satisfied with their roster (range: 0–100).

I am very happy with the rostering on my ward although I only work 20–30 h per fortnight there is always the opportunity to pick up extra shifts if I can do them. (Nurse and midwife survey respondent)

Most respondents (*n* = 444, 78.3%) agreed that having more flexible roster guidelines would improve their satisfaction with their roster (Supplementary Material 1: Nurse and midwife survey data - Table 6).

There aren’t many positive aspects. The ability to swap once you have found someone to swap with is easy - just an email to line manager. But otherwise it’s unfair and not the same across the board. (Nurse and midwife survey respondent)

##### Personal leave

More than half of the respondents stated that they had taken personal leave in the previous six months due to their roster resulting in fatigue (*n* = 335, 59.1%) or because their roster requests were unable to be met (*n* = 310, 54.7%) (Supplementary Material 1: Nurse and midwife survey data - Table 7).

[The roster] is not flexible & fair - that is probably why people call sick or eventually leave. (Nurse and midwife survey respondent)

The lack of consistency [in roster] means we are always fatigued and sleep poorly. (Nurse and midwife survey respondent)

##### Additional shifts

More than half the respondents (*n* = 373, 58.2%) said they were contacted about 1–4 times in an average week to do additional shifts, and over two-thirds (*n* = 379, 67.1%) were asked to work additional shifts when they had not stated they were available. Most respondents were contacted via social media (*n* = 257, 36.3%) or SMS (*n* = 309, 43.6%) to do additional shifts (Supplementary Material 1: Nurse and midwife survey data - Table 8).

##### Shift swaps

Although most respondents (*n* = 470, 75.9%) stated that there was an agreed process for swapping shifts in their ward/unit, over two-thirds (*n* = 416, 67.2%) were approached at least once or twice a fortnight to swap a shift mostly by a colleague (*n* = 473, 76.4%) and almost half (*n* = 254, 44.9%) reported no or limited flexibility in their ward/unit if they needed to change their shift on a particular day. The respondents were asked how easy it was to swap shifts on a scale ranging from ‘very difficult’ (0) to ‘very easy’ (100) the mean score was 46.5 indicating that overall, the respondents found it difficult to swap shifts. Using a similar scale, respondents were asked the percentage of time they had been successful in swapping shifts over last the last six months and the sample’s mean score was 53.2 indicating that respondents were not always successful having their requests fulfilled (Supplementary Material 1: Nurse and midwife survey data - Table 9).

[The roster is] not often flexible, and hard to find swaps. (Nurse and midwife survey respondent)

MUM’s are very accommodating when need to be. However, the whole rostering process is one of the worst I have worked with in terms of having requests denied/ignored etc. (Nurse and midwife survey respondent)

##### Redeployment

The respondents were asked to rate whether they thought redeployment was managed in a fair manner in their ward/unit on a 100-point scale which ranged from ‘totally unfair’ (0) to ‘totally fair’ (100). The mean score of all the respondents was 34.6 (range 0–100) indicating that most respondents thought the redeployment process was unfair.

The respondents wrote many comments about redeployment. They stated that they understood why redeployment was required particularly to ensure an adequate skill mix but they also reported that they were often redeployed without warning especially at the start of a shift; they perceived that it was usually the same people who were redeployed; they often felt unprepared for redeployment; and redeployment decreased their work satisfaction and was stressful.

A lot of the time the same people are moved on the day of or even minutes before starting a shift including myself because we do not complain. Certain people complain about working in an area and others get moved from that area to accommodate for outspoken people. There are staff that will not be moved and it is very unfair. (Nurse and midwife survey respondent)

##### Night shifts

Most respondents (*n* = 428, 75.5%) said that they would like to have the ability to choose when they worked night shift as this would improve their satisfaction with the roster and would prefer to do night shift in a block (*n* = 437, 77.1%). Almost a third (*n* = 164, 28.9%) stated that they would prefer not to work night shifts and although a small proportion would prefer to work permanent night duty, most nurses and midwives surveyed indicated that the option of permanent night duty would not suit them (*n* = 472, 83.2%) (Supplementary Material 1: Nurse and midwife survey data - Table 10).

Having to do night shifts every 5–6 weeks for 2 weeks can really take a mental toll. (Nurse and midwife survey respondent)

##### Important roster factors

Respondents were asked to rate on a scale ranging from ‘least important’ [1] to ‘most important’ [9] the importance of several factors in their roster. Factors identified as important by the respondents included the ability to self-roster, swap shifts and negotiate their roster; equity for all staff; and having an even skill mix across shifts (Supplementary Material 1: Nurse and midwife survey data - Table 11).

##### Nurse and midwife comparison

Almost two-thirds of the survey respondents reported that they were working as nurses (*n* = 356, 63.2%) and just over a third were midwives (*n* = 207, 36.8%).

Over a third of nurses (*n* = 126, 35.4%) had an informal flexible work arrangement compared to about 15% (*n* = 33) of midwives (*p* < 0.001) but a similar proportion of nurses (*n* = 38, 10.7%) and midwives (*n* = 21, 10.1%) had a formal flexible work arrangement.

Midwives were significantly more likely than nurses to report having taken personal leave in the previous six months due to their roster resulting in fatigue (66.2% vs. 55.1%; *p* = 0.010) or because their roster requests were unable to be met (64.3% vs. 49.2%; *p* < 0.001); being contacted about 1–2 times in an average week to do additional shifts (51.1% vs. 46.2%; *p* < 0.001); being asked to work additional shifts when they had not stated they were available (81.6% vs. 58.4%; *p* < 0.001); perceive there was no flexibility in their ward/unit if they needed to change their roster on a particular day (32.4% vs. 13.8%; *p* < 0.001); it was difficult to swap shifts (42.4 vs. 48.7; *p* = 0.003); being redeployed at least once per week to a different area on the day of their shift (30.9% vs. 4.8%; *p* < 0.001). Midwives were also significantly more likely than nurses to report that having the ability to swap shifts if required (Mean score: 6.5 vs. 5.9; *p* < 0.001) and the ability to negotiate their roster (Mean score: 5.1 vs. 5.0; *p* = 0.022) were important factors in their roster.

Midwives were significantly less likely than nurses to report that their roster requests were usually supported (37.2% vs. 45.8%; *p* < 0.001); they were satisfied with their roster (Midwife mean score 48.1 vs. nurse 62.3; *p* < 0.001); and that they were usually not successful in having their shift swap requests fulfilled (50.4 vs. 54.8; *p* = 0.047).

#### Unit manager survey

Fourteen unit managers completed a survey. Most respondents (*n* = 10, 71.4%) were aged in their forties and fifties, and had been a nurse or midwife for about 10–20 years (*n* = 10, 71.4%) and a unit manager for five or less years (*n* = 8, 57.2%), and reported that they spent about 80% of their time doing non-clinical work (*n* = 11, 78.6%) (Supplementary Material 2: Unit and roster manager survey data - Table 1).

##### Roster practices

Over half of the respondents (*n* = 8, 57.1%) reported that they supervised the writing of the roster in their area and spent on average about five hours writing or reviewing the roster and one hour each day reworking it. Over half of the respondents (*n* = 8, 57.1%) indicated that they had staff with flexible work arrangements and most thought that formal flexible work arrangements had a negative impact on compiling the roster (*n* = 9, 64.3%) (Supplementary Material 2: Unit and roster manager survey data - Table 2).

##### Roster requests

All respondents (n = 14, 100%) reported that they had a roster request system in their ward/unit and felt that their staff usually got the shifts they requested (n = 14, 100%). Most respondents (n = 12, 85.7%) had to contact nurses/midwives in their area 1–2 times in a 24-hour period to pick up additional shifts. Using a scale which ranged from ‘very difficult’ (0) to ‘easy’ (100), most respondents felt that it was mostly easy for nurses/midwives to swap shifts (mean score = 70.9, SD = 15.9). Respondents also felt that giving nurses/midwives more control over their roster would increase their satisfaction (mean score = 81.1, SD 25.2; 100 point scale ranging from ‘totally disagree’ (0) to ‘totally agree’ (100)). Most respondents (n = 11, 78.6%) thought that current rosters resulted in nurses/midwives taking personal leave due to fatigue and to accommodate their caring responsibilities if their roster requests were not met (n = 13, 92.9%). Respondents (n = 6, 85.7%) mostly reported that they contacted nurses/midwives to work additional shifts even when the nurses/midwives had not stated they were available to work a particular additional shift but all respondents (n = 14, 100%) reported that they tried to be flexible in accommodating nurses’/midwives’ requests to change their rostered hours on a particular day. The respondents also felt that redeployment on the day of a shift was managed in a fair manner in their ward/unit (mean score = 72.5, SD 4.4; 100-point scale ranging from ‘0’ totally unfair to ‘100’ totally fair).

#### Roster manager survey

Thirteen roster managers completed a survey. More than half the respondents (*n* = 7, 53.9%) had been a roster manager for more than three years and undertook the role on a permanent basis (*n* = 11, 84.6%) (Supplementary Material 2: Unit and roster manager survey data - Table 1).

##### Roster practices

Most respondents (*n* = 11, 84.6%) reported that they prepared the roster for their unit/ward and then the NUM/MUM approved it. On average the respondents stated that they spent about 5 h per week writing the roster and about 2.5 h per day reworking it. Just over half of the respondents (*n* = 7, 53.9%) felt they had enough time to complete the roster. About two thirds of the respondents (*n* = 8, 61.5%) indicated that they had staff with flexible work arrangements and almost half felt these had a negative impact on compiling the roster (*n* = 6, 46.2%).

##### Roster requests

All respondents (*n* = 13, 100%) stated there was a roster request system in their ward/unit and they had a good understanding of their staff’s roster preferences (*n* = 13, 100%). Most respondents (*n* = 12, 92.3%) felt that the nurses/midwives in their area usually or always got the shifts they requested and agreed that giving nurses/midwives more control over their roster would increase their satisfaction (mean score = 77.4, SD 20.9; 100 point scale ranging from ‘totally disagree’ (0) to ‘totally agree’ (100)) and that staff often took personal leave due to roster related fatigue (*n* = 6, 46.2%) or to manage their caring responsibilities (*n* = 9, 69.2%).

Try to always grant 100% of requests where possible whilst ensuring fair & equitable roster with balanced cover. (Roster manager survey respondent)

In terms of type of shift preferences I will generally try to aim for majority/ 60% of their roster being their preferred shift. Not at the expense of skill mix though… I would rather they feel good about coming to work, that they haven’t had to ask for a million shift swaps …They’ll be more likely to do their EFT if they get the shifts they need, they’ll be more likely to pick up shifts that were short if it fits in with them. … Incorporate staff wishes and lifestyle as much as possible. (Roster manager survey respondent)

### Component 2 (focus groups)

Thirty-nine nurses, midwives and unit and roster managers across the three study health services participated in a focus group. Nine focus groups were held in total and the number of participants in each group ranged from three to seven.

#### Nurse and midwife focus groups

Fourteen nurses from the study health services participated in five focus groups and nine midwives from the two study health services which provide maternity services participated in two focus groups. The nurse and midwife participants were mostly aged between 25 and 49 years (*n* = 17, 73.9%) and had been a nurse or midwife for more than ten years (*n* = 12, 52.2%).

The analysis of the transcripts from the five focus groups with nurses and two with midwives identified two main themes: (1) nurses’ and midwives’ concerns about current roster practices and guidelines; and (2) the impact of current rostering practices on their work and personal lives and health. Within each theme, subthemes were also identified.

#### Theme 1: nurses’ and midwives’ concerns about current roster practices and guidelines

##### Perceptions of fairness and equity

The participants reported several concerns about the current roster practices and guidelines in their area. These included perceptions about the fairness and equity of the roster and flexible work arrangements. These perceptions were often related to the participant’s life stage particularly whether they had children. Many participants commented that people with (young) children were often given preferential treatment in terms of roster requests and flexible work arrangements especially compared to people who do not have children. Participants who did not have children often perceived that when they did eventually have children, they would not necessarily receive similar treatment.

There’s lots of people with young children, and fully supportive and understanding that they have requirements … but it seems their requests are always granted. (Focus group participant - nurse)

I was rostered every Saturday and Sunday for 2 months straight, … I get quite angry … I might not have kids, but I still have family I want to see, I still have friends I want to see … we kind of get pushed to the end of the queue. (Focus group participant - nurse)

Participants felt that current roster allocations were often unfair as they perceived roster managers gave ‘their friends’ the preferred shifts and it was difficult to get preferred shifts after returning from parental leave. Participants believed that more transparency about how shifts were allocated was required.

There should be a lot more equity in [the roster] and a lot more sharing of the load. Because not everybody wants to [work] those harder to do days. (Focus group participant - midwife)

When flexible working arrangements are given to some but not to everybody I think that’s a fairness issue. (Focus group participant - midwife)

##### Roster requests not supported

Another concern many focus participants reported was that their roster requests were often not supported. They said unsupported roster requests were frustrating, had a negative impact on their work life balance or ability to manage their other commitments, and meant they had to find someone to swap shifts with or take personal leave which had an adverse effect on their entire team.

Some months I got absolutely zero requests … which makes it really difficult when you’ve got childcare. And the solution always was just see if you can swap. (Focus group participant - midwife)

I have to call in sick [if roster request not supported], use my personal leave hours and that leads to the ward being short-staffed because they can’t fill that vacancy so then it affects not just me but everyone else on the ward … and the patient care gets neglected. (Focus group participant - nurse)

##### Difficulties managing other commitments and responsibilities

The participants reported difficulties managing their personal responsibilities and commitments within the current roster system. They perceived this was mainly due to limited flexibility and an inability to have advanced notice about their roster within the current system.

Our child care centre is … about 45 min from the hospital, and doesn’t open in time for you to get to early shifts. (Focus group participant - midwife)

You want the roster out as much in advance as possible so you can plan around it and have your life and have a little bit of balance, but then the other side to that is if something pops up short notice, there’s very little flexibility to find someone to cover your shift, and then not put other people out. (Focus group participant - nurse)

Most of us don’t even have commitments anymore because we can’t make the commitment … we have to work our commitments around our roster that changes every month, every fortnight. (Focus group participant - nurse)

#### Theme 2: impact of current roster practices on work and personal lives and health

Participants also discussed the impact of current rostering practices on various aspects of their work and personal lives and health including their ability to perform their duties, turnover intentions and the often-adverse impact of the roster on their health.

##### Difficulties providing high quality patient care

Participants commented that their current rosters often caused fatigue which negatively affected their ability to provide high quality patient care and increased the likelihood of errors.

If I do a late shift I’ll usually get home around 10.30 … By the time you destress, chill out and go to sleep, you’re only getting 3–4 h sleep … before you’ve got to be up at 5, out to work again … on my ward you’ve got to think clearly about what you’re doing … if I miss something, it can easily cause quite a problem … you’ve got to be sharp, you have to be really alert. (Focus group participant - nurse)

I struggle at the moment [with] a very frequent rotation of night shift blocks … there’s no time to recover, and I feel then when I’m at work I’m either sleep deprived, or drained and not then able to work effectively. (Focus group participant - nurse)

##### Adverse health effects

The participants made many comments about the adverse health effects of their rosters and shift work including fatigue, burnout, car accidents, and limiting their ability to exercise.

There’s lots of fatigue and the inability to like changing back your circadian rhythm to the day shift after night shift. Yeah, definitely a lot of health impacts in that sense, causing a lot of sick leave and things of the sort. (Focus group participant - midwife)

You are so tired you are not really alert … trying to go from being awake during the day time and then the next few days being awake during the night time … there’s a negative impact on our wellbeing and our mental health. (Focus group participant - nurse)

Many participants stated that they often took personal leave because of their roster schedule, their roster requests or shift swaps not being supported, or roster related fatigue and burnt out.

I personally have called in sick because [I was] not coping and I felt like my requests were just not heard, even though I’ve requested this, I flagged it as an issue, it just continuously happens. (Focus group participant - nurse)

There’s a certain amount of people who would call in sick, they go I just can’t do that… and if you have a horror night or a couple of delirious patients sometimes you’ll sleep like rubbish because you’ve had a terrible night and you’re like oh my god I couldn’t possibly come back. (Focus group participant - nurse)

##### Negative impact of roster on job satisfaction and turnover intentions

The participants discussed the impact of current roster practices and shift work on their job satisfaction and turnover intentions. Several reported that a lack of flexibility with their roster or inability to work preferred shifts meant that they had considered leaving their health service and nursing/midwifery.

I’ve probably thought about [leaving midwifery] multiple times, it’s certainly complex - there’s many facets to it, but it comes down to often feeling undervalued and that it, as long as there’s a name on the roster it doesn’t matter. (Focus group participant - midwife)

It’s a lot of late/earlies or a lot of chopping and changing of shifts and … I’ve also had the random night shifts in amongst my AM and PMs and it’s draining, … especially when you’re like six months [into your graduate nurse] program and you’re already feeling burnt out, it’s like how do you sustain a career at that point. (Focus group participant - nurse)

It’s hard to think about leaving a job that you genuinely love doing because somewhere else would be more flexible. (Focus group participant - nurse)

##### Unit and roster manager focus groups

Sixteen unit and roster managers participated in two focus groups. The participants were mostly aged between 25 and 49 years (*n* = 10, 62.5%) and had been in their current role for less than five years (*n* = 12, 75.0%). The participants discussed their thoughts and experiences of current roster practices in their health service. The analysis of the focus group transcripts identified one main theme about challenges with roster preparation and implementation.

#### Theme 1: challenges with roster preparation and implementation

##### Ensuring fairness and equity

The participants reported that they attempted to ensure fairness and equity with the rosters in their areas but recognised that it was often the same people who did additional shifts and they had to manage expectations about who should work when.

I’ve got regular people that will often be very flexible … they’ll just pick up shifts here there and everywhere, but I worry about them most and there was an error on nightshift for a staff member who had done this. (Focus group participant – roster/unit manager)

I think the established staff feel like they’re actually owed … they’ve worked really really hard so why shouldn’t they get the Friday evening off, like it’s the young people’s turn to do that. (Focus group participant – roster/unit manager)

It is a challenge to balance the roster on PMs and nights and weekends and make it fair for all. (Focus group participant – roster/unit manager)

##### Difficulties filling the roster and accommodating staff preferences

The unit and roster managers identified difficulties they experienced completing the rosters including that particular shifts were often difficult to fill which negatively impacted their ability to ensure an adequate skill mix. They also reported having to remind staff that nursing and midwifery are 24 h a day / 7 days a week roles and it was expected that they would be able to work all shifts.

It seems like nobody wants to do nights ever, so that seems to be my hardest thing … just trying to get nights fair and every person thinks that they do nights too frequently. (Focus group participant – roster/unit manager)

I suppose the challenge that we’ve come across is probably skill mix, so now there’s more junior staff, that means we’re having to take the more experienced staff off the shifts they want and fill them with junior staff so that’s kind of changed the dynamic a bit and probably resulted in a few more complaints. (Focus group participant – roster/unit manager)

The unit and roster managers discussed challenges trying to accommodate staff’s shift and roster preferences while at the same time ensuring there was an adequate skill mix, the rosters were compliant with the enterprise agreement, and the health service could operate. The participants reported that they attempted to satisfy nurses’/midwives’ roster requests as this minimised unplanned personal leave. In general, the participants felt staff in their areas were satisfied with their rosters.

I try to be as flexible as possible too, and people – once the roster is out often people will come to me and I will be flexible and swap their shifts otherwise they’ll just call in sick. (Focus group participant – roster/unit manager)

For maternity a lot of midwives have preferences as to where they want to work, some midwives want to work in birthing as opposed to others who only want to work on the post-natal wards … you don’t want to create a very rigid workforce that de-skills because staff aren’t able to work across the child-bearing continuum but at the same time if staff are working where they want to work then they’re more likely to turn up for the shift. (Focus group participant – roster/unit manager)

##### Assist staff to manage other responsibilities

The participants reported that they attempted to support staff manage their other responsibilities and commitments within current roster practices and guidelines as this assisted with staff satisfaction.

We’ve got a lot of working mums … so it’s always a constant juggle to do what we can to accommodate their requests as much as we possibly can. (Focus group participant – roster/unit manager)

We find that once the roster is published people – things come up in personal life and they need to make changes, so we’re really onboard with just making sure that staff can make these changes, so we’ll facilitate those changes as best as we can to keep the place operational. (Focus group participant – roster/unit manager)

### Component 3 (co-design workshops)

Overall, 91 nurses and midwives participated in the four co-design workshops (one at each participating health service and two at health service #3) and of these 40 completed the demographic survey. Most participants were nurses (*n* = 32, 80.0%) and on average were aged 40.4 years, had practised as a nurse or midwife for 14.2 years and had worked at their current health service for 8.0 years (Supplementary Material 3: Co-design workshops - Table 1).

The participants identified several preferred roster initiatives and practices. These were mainly about ensuring flexibility, fairness and equity, and fatigue management especially in relation to night shift, roster requests, shift start/finish times and lengths, redeployments and rotations (for midwives), and roster resources and guidelines.

#### Flexibility

The participants stated that nurses and midwives would appreciate flexibility with their rosters including the ability to self-roster and have different shift start/finish times and lengths as this would enable them to better manage their other commitments and responsibilities such as being able to drop off and pick up children from childcare. Many midwives felt there should be more flexibility about which areas they worked in, and many would appreciate the option to work solely in their ‘core’ or preferred area. The participants perceived that this would assist with increasing retention, job satisfaction, quality of patient care and decrease personal leave.

I personally think more flexible start and finish times and lengths would be helpful as it would mean I would be potentially available for more shifts and provide a better work/life balance. Arranging before school care/after school care + an early morning nanny for my kids is a juggle and quite costly. (Co-design workshop participant)

The same [roster] rules don’t work for every single person. (co-design workshop participant)

I’m personally also interested in limiting rotations and midwives having more flexibility in choosing the area/ areas they wish to work in. … Working in the area(s) you enjoy and are passionate about would increase job satisfaction and retention of staff whilst decreasing personal leave calls and ensuring better care to women. (Co-design workshop participant)

#### Fairness and equity

Most participants identified a need for fair and equitable roster guidelines and practices particularly about roster requests, working ‘unpopular’ shifts such as weekend and night shifts, and redeployment/reallocation. The participants suggested that all nurses and midwives should be guaranteed a certain proportion of their roster requests, and night shift and redeployment planners should be implemented to ensure the same people did not always work night shift or were redeployed. They perceived that such initiatives would ensure fairness and equity, support individuals’ roster preferences, and minimise the need for nurses and midwives to take personal leave.

I think getting “guaranteed” shifts based on your EFT is a great idea. And people are less likely to be unhappy about the unguaranteed/ or allocated shifts for the remainder of their shifts. I know lots of people who put in shift requests now and often don’t get them. (Co-design workshop participant)

It would be nice to have the option to be core in a specific area rather than rotational, some people have anxiety working in certain areas. Or if core is not an option, then I think they need to rotate everyone more fairly. Some people end up in the same areas for months at a time which then creates more anxiety around going to different areas. (Co-design workshop participant)

#### Fatigue

The participants discussed roster practices and guidelines which would assist nurses and midwives to manage fatigue including appropriate night shift frequency, shift block lengths and (consecutive) days off.

I mentally struggle with nights so having a full fortnight of them for me is draining. Whereas 1 a week for me is very doable. (Co-design workshop participant)

I have had nights followed by days with hardly any time to turn your body clock around and it made me ill. For example, working 4 nights, finishing on the Monday morning, having Tuesday as my full day off and being back on 3 × 12 h day shifts after 1 full day to turn myself around was no where near enough. I was exhausted and unwell for my days off. (Co-design workshop participant)

As for days off after nights personally I need a sleep day off and an additional day to feel ready to come back to days again. (Co-design workshop participant)

#### Roster practices and education

The participants reported that it would be beneficial to have easily accessible information and education about roster guidelines and practices. This could include a resource for new or graduate staff so they can become familiar with the roster practices in their area; and more support and training for roster managers particularly about roster guidelines, appropriate enterprise bargaining agreements and legislation, and communication skills training.

The roster manager should get proper training and on going education about guidelines in regards to rostering. (Co-design workshop participant)

## Discussion

This study aimed to explore nurses’ and midwives’ experiences and perceptions of current roster guidelines and practices and identify their preferences for acceptable and feasible principles. The findings indicate that although nurses and midwives were mostly satisfied with their rosters, they often experienced frustrations and challenges with current roster guidelines and practices as well as adverse effects on their health and work and personal lives. Nurses and midwives identified a preference for fair and equitable rosters which provide flexibility and enable them to manage their other commitments and responsibilities and reduce roster-related fatigue. Nevertheless, the findings suggest that a ‘one size fits all’ approach to roster guidelines and practices does not suit the current nursing and midwifery workforce. As one participant commented ‘the same rules don’t work for every single person’.

### Flexibility

Inflexible schedules have been identified as a serious psychosocial hazard [43]. As found in this study, inflexible roster practices and guidelines often made it difficult for nurses and midwives to achieve their work preferences (e.g. preferred shifts) and manage their paid work and personal lives including caring responsibilities and education and sporting commitments. Offering flexible work schedules is one way health services can attract and retain their nursing and midwifery workforce [44]. Other studies have found that greater work schedule flexibility is associated with lower levels of emotional exhaustion and burnout among nurses [5, 45, 46], and higher levels of job satisfaction [46] and patient safety [47].

The findings of this study and others indicate that not only do nurses and midwives desire more flexibility and control over their roster, they also want roster practices that consider their individual needs. Flexible workplaces have benefits for both nurses and midwives and their employing health services including improved employee satisfaction and wellbeing, work/life balance, patient outcomes, and reduced turnover and absenteeism [44, 45, 47–49].

### Fairness and equity

Generating rosters can be complex and it is often difficult to accommodate the needs and preferences of individual nurses and midwives as well as ensure appropriate staffing levels and skill mix. A recent review of 46 publicly available health service roster policies found that although all included an objective about roster fairness, none explicitly defined fairness or provided a framework about how fairness was to be achieved [50]. The unit and roster managers who participated in this study overwhelmingly stated that they attempted to manage roster requests in a fair and equitable manner and felt it was easy for nurses and midwives to swap their shifts. Yet many nurse and midwife participants felt that roster practices particularly roster requests and redeployments in their area were not fair and equitable. They reported frustration and resentment when they perceived that the same nurses or midwives always had their requests fulfilled or were reallocated at the beginning of a shift.

Although little is known about the impact of perceived favouritism and lack of equity in the preparation and scheduling of nurse and midwife rosters, similar to the findings of this study, other research has highlighted an association with these factors and health service staff’s job dissatisfaction, lower quality of patient care and higher turnover intentions [51, 52]. The findings of this study and others indicate that roster practices and guidelines should aim to be fair and equitable, in that, they should consider individual needs, preferences and differences. A ‘one size fits all’ approach appears to be simplistic and unlikely to promote nurse and midwife wellbeing and job satisfaction [53].

### Fatigue management

Shift work including night shifts, inadequate breaks between shifts, shift start and finish times, and the length of shifts can contribute to occupational fatigue among nurses and midwives [9, 54, 55]. Fatigue can have adverse effects for nurses, midwives and patients including increased risk of clinical and medication errors [9, 54–57], accidents and injuries [9, 55, 58]; poor communication [59]; compassion fatigue [60]; job dissatisfaction [61]; lack of concentration [54]; and physical and mental health conditions such as heart disease, musculoskeletal disorders, obesity, and depression and anxiety [62–64].

Over half of the nurses and midwives in this study reported that they had taken personal leave in the last six months due to their roster resulting in fatigue. Consistent with the findings of others [9, 55], the nurses and midwives in this study wanted rosters which reduce roster-related fatigue and ensure adequate rest between shifts. Measures such as modifying the number of consecutive night shifts and the sequencing of days off between shifts and reducing ‘late earlies’ / ‘quick returns’ (night to day transitions) were suggested by the participants in this study and have been shown by others to reduce some of the negative effects of shift work [61, 64–68].

### Foundations

Rostering can be a difficult task. This study has highlighted the considerable time unit and roster managers spend creating and revising rosters for their area/ward, and the frustrations they and nurses and midwives often have with current roster systems. As acknowledged by others [50, 69, 70] and consistent with the findings of this study, nurse and midwife unit and roster managers identified a need for acceptable, user-friendly, and efficient rostering systems that enable them to prepare rosters which are flexible, fair and equitable, ensure adequate staffing levels and skill mix, and take into consideration health service and regulatory policies, frameworks and legislation. They also recognised the need for adequate allocated time and education to prepare rosters.

### Strengths and limitations

This study was conducted as a rapid response to sector concerns about nursing and midwifery workforce and wellbeing challenges. The study used mixed methods including co-design to comprehensively investigate the roster experiences, perceptions, and preferences of a range of nurses and midwives employed in public health services in metropolitan and regional settings. Despite a high response rate, only three health services providing mostly acute care in one state of Australia were included and therefore, the findings may not be generalisable to other settings. A strength of this study was the inclusion of nurse and midwife unit and roster managers as well as ward-based nurses and midwives. This enabled a wider examination of nurse and midwife roster principles, practices, and preferences. The trustworthiness of the qualitative data (focus groups and co-design workshops) was assured by discussions amongst the research team to ensure consensus about the themes identified, the inclusion of three diverse health services and a range of different data collection techniques. The focus groups and co-design workshops were facilitated by members of the research team who were not employed at the participating health services (i.e. they were employed by a university partner and government health agency) and unknown to the participants in order to reduce the possibility of social desirability bias.

### Implications for nursing and midwifery policy and practice

The findings of this study have implications for current nursing and midwifery roster practices and principles. The findings indicate that roster practices and principles should take into consideration nurses’ and midwives’ individual needs and preferences and incorporate flexibility, fairness and equity, and fatigue management. Based on these findings, the research team has developed a resource, the Victorian Rostering Toolkit [71], for nursing and midwifery unit managers and roster managers outlining employee-centred rostering principles. The Toolkit is designed to assist health services implement consistent and best-practice local rostering guidelines. Nonetheless, health services should continuously monitor, evaluate, and revise their rostering principles and practices to ensure they are compliant with legislative frameworks, meeting staff needs and promoting best care.

### Future research

The co-design workshops conducted in Component 3 of this study identified possible roster initiatives for each of the participating health service. The next stage of this research is to evaluate the feasibility and acceptability of the implemented initiatives. Further research conducted in other settings such as private hospitals, aged care and community settings would also further understanding of nurses’ and midwives’ experiences of and preferences for roster guidelines.

## Conclusion

The findings of this study indicate that nurses and midwives often experience frustrations and challenges with current roster guidelines and principles as well as adverse impacts on their health, and work and personal lives. A ‘one size fits all’ approach does not appear to meet the needs and preferences of the current nursing and midwifery workforce. Nurses and midwives have identified a preference for fair and equitable rosters which provide flexibility and enable them to manage their other commitments and responsibilities, reduce roster-related fatigue, and provide high quality patient care.

## Electronic supplementary material

Below is the link to the electronic supplementary material.

Supplementary Material 1

Supplementary Material 2

Supplementary Material 3

Supplementary Material 4

Supplementary Material 5

Supplementary Material 6

Supplementary Material 7

Supplementary Material 8

## Data Availability

Data is provided within the manuscript or supplementary information files.
