# Supplementary material for: Developing nurse and midwife centred rostering principles using co-design: a mixed-methods study
Source: BMC Nurs. 2024 Dec 20;23:938. doi: 10.1186/s12912-024-02522-7 (PMC11660556; doi:10.1186/s12912-024-02522-7)
Supplement: Supplementary file 1 — Supplementary Material 1 [file 12912_2024_2522_MOESM1_ESM.docx]

**Supplementary Material 1**

**Component 1: Nurse and midwife survey data**

**Table 1: Respondent employment and sociodemographic characteristics**

| **Characteristic (n, %)** | **Total**  **N=688** |
| --- | --- |
| Role (n, %) |  |
| ANUM/AMUM | 118 (17.2) |
| GNP/GMP | 42 (6.1) |
| RN/RM/CNS/CMS/EN | 514 (74.7) |
| Other | 14 (2.0) |
| Employment Status |  |
| Full time | 103 (15.0) |
| Part time | 585 (85.0) |
| Hours worked per fortnight |  |
| Mean (SD), range | 54.3 (15.7), 0.4-106.0 |
| Age |  |
| <21 | 4 (0.6) |
| 21-25 | 132 (19.2) |
| 26-30 | 163 (23.7) |
| 31-35 | 116 (16.9) |
| 36-40 | 91 (13.2) |
| 41-45 | 52 (7.6) |
| 46-50 | 51 (7.4) |
| 51-55 | 36 (5.2) |
| 56-60 | 22 (3.2) |
| >60 | 21 (3.1) |
| Years worked as a nurse |  |
| < 1 year | 57 (8.3) |
| 1-2 years | 75 (10.9) |
| 3-5 years | 126 (18.3) |
| 6-10 years | 114 (16.6) |
| 11-20 years | 110 (16.0) |
| > 20 years | 96 (14.0) |
| Years worked as a midwife | 109 (15.9) |
| < 1 year | 33 (4.8) |
| 1-2 years | 48 (7.0) |
| 3-5 years | 69 (10.1) |
| 6-10 years | 45 (6.6) |
| 11-20 years | 45 (6.6) |
| > 20 years | 24 (3.5) |
| Years worked at current health service |  |
| < 2 years | 228 (33.1) |
| 3-5 years | 203 (29.5) |
| 6-10 years | 121 (17.6) |
| 11-20 years | 85 (12.4) |
| > 20 years | 51 (7.4) |
| Clinical work area |  |
| Medical | 145 (27.9) |
| Surgical | 78 (15.0) |
| Subacute nursing | 41 (7.9) |
| Emergency | 45 (8.7) |
| Midwifery | 207 (39.8) |
| Coronary care services | 4 (0.8) |
| Rotate through more than one area of speciality (‘yes’ responses) | 174 (30.9) |
| Rotation length |  |
| 1-2 shifts per week | 68 (39.1) |
| 3-4 shifts per week | 31 (17.8) |
| 1-3 week block | 35 (20.1) |
| Greater than 4 weeks | 40 (23.0) |

**Roster variables**

**Table 2: Roster arrangements**

| **Characteristic (n, %)** | **Total**  **N=688** |
| --- | --- |
| Work rotating roster (7-day week) (‘yes’ response) | 589 (89.4) |
| Work rotating roster due to role (‘yes’ response) | 43 (61.4) |
| Days worked |  |
| Weekdays only | 30 (4.6) |
| Weekdays and weekends | 621 (94.2) |
| Weekends only | 8 (1.2) |
| Shifts worked |  |
| Days | 596 (84.2) |
| Afternoons | 580 (81.9) |
| Nights | 548 (77.4) |
| Business hours | 40 (5.6) |
| Informal flexible work arrangements (‘yes’ response) |  |
| Yes | 189 (28.7) |
| Formal flexible work arrangements (‘yes’ response) |  |
| Yes | 73 (11.1) |

**Table 3: Respondents’ other responsibilities and commitments**

| **Characteristic (n, %)** | **Total**  **N=688** |
| --- | --- |
| Have responsibilities/commitments which impact roster availability (‘yes’ response) | 406 (61.6) |
| Type of responsibility/commitment |  |
| Childcare | 201 (28.4) |
| Care for others | 141 (19.9) |
| Education | 77 (10.9) |
| Personal health care | 95 (13.4) |
| Volunteering | 29 (4.1) |
| Work elsewhere | 58 (8.2) |
| Other | 54 (7.6) |

**Table 4: Roster guidelines and request system**

| **Characteristic (n, %)**  *(‘yes’ responses)* | **Total**  **N=688** |
| --- | --- |
| Ward/unit has roster guidelines for staff | 202 (30.6) |
| Roster guidelines easily accessible | 119 (58.9) |
| Ward/unit has rostering request system | 608 (98.1) |

**Table 5: Roster requests**

| **Characteristic (n, %)** | **Total**  **N=688** |
| --- | --- |
| Average number of roster requests submitted in a fortnight |  |
| All shifts | 94 (14.7) |
| 0-1 shifts | 287 (44.8) |
| 2-5 shifts | 208 (32.4) |
| >5 shifts | 52 (8.1) |
| Roster request supported |  |
| Never | 25 (3.9) |
| Rarely | 61 (9.5) |
| Occasionally | 173 (27.0) |
| Usually | 274 (42.7) |
| Always | 108 (16.8) |

**Table 6: Roster satisfaction**

| **Characteristic (n, %)** | **Total**  **N=688** |
| --- | --- |
| Level of satisfaction with roster (100-point scale: 0 ‘not satisfied’ to 100 ‘satisfied’) |  |
| Mean, range | 57.4, 0-100 |
| Would more flexible rostering guidelines improve work schedule (‘yes’ response) | 444 (78.3) |
|  |  |

**Table 7: Personal leave**

| **Characteristic (n, %)**  *(Taken personal leave in previous 6 months; ‘yes’ responses)* | **Total**  **N=688** |
| --- | --- |
| Due to roster resulting in fatigue | 335 (59.1) |
| Due to roster requests not met | 310 (54.7) |

**Table 8: Additional shifts**

| **Characteristic (n, %)** | **Total**  **N=688** |
| --- | --- |
| Number of times contacted to do additional shift in average week |  |
| 0 | 166 (25.9) |
| 1-2 | 272 (42.4) |
| 3-4 | 101 (15.8) |
| 5-6 | 35 (5.5) |
| >6 | 67 (10.5) |
| How contacted |  |
| Phone call | 209 (29.5) |
| SMS | 309 (43.6) |
| WhatsApp | 20 (2.8) |
| Social media | 257 (36.3) |
| Other | 94 (13.3) |
| Number of PM shifts followed by AM worked in fortnight |  |
| 0 | 135 (21.5) |
| 1-2 | 321 (51.2) |
| 3-4 | 156 (24.9) |
| 5-6 | 15 (2.4) |
| Asked to work additional shifts when haven’t stated available | 379 (67.1) |

**Table 9: Shift swaps**

| **Characteristic (n, %)** | **Total**  **N=688** |
| --- | --- |
| Agreed process for swapping shifts in ward/unit | 470 (75.9) |
| No or limited flexibility on ward/unit if have legitimate need to change roster on particular day | 254 (44.9) |
| How easy to swap shifts (100-point scale: 0 ‘very difficult’ to 100 ‘very easy’) |  |
| Mean, range | 46.5, 0-100 |
| Percentage of time successful in swapping shifts over last 6 months (100-point scale: 0% to 100%) |  |
| Mean, range | 53.2, 0-100 |
| How often approached to swap shift in a fortnight |  |
| 0 | 117 (18.9) |
| 1-2 | 416 (67.2) |
| 3-4 | 61 (9.9) |
| 5-6 | 13 (2.1) |
| >6 | 12 (1.9) |
| Who approached by to swap shifts |  |
| Manager | 19 (3.1) |
| Colleague | 473 (76.4) |
| Both | 127 (20.5) |

**Table 10: Night shift**

| **Characteristic (n, %)** | **Total**  **N=688** |
| --- | --- |
| Ability to choose when rostered to night shifts would improve roster satisfaction (‘yes’ response) | 428 (75.5) |
| How often would like to do night-duty in 12-month period |  |
| Never | 164 (28.9) |
| 2 weeks a year | 63 (11.1) |
| 4 weeks a year | 127 (22.4) |
| 8 weeks a year | 124 (21.9) |
| Greater than 8 weeks | 89 (15.7) |
| Would like to complete night shift in block or mixed with day & afternoon shifts |  |
| One block | 437 (77.1) |
| Mixed with other time shifts | 130 (22.9) |
| Would option of permanent night duty suit you (‘no’ response) | 472 (83.2) |

**Table 11: Important roster factors**

| **Characteristic (n, %)**  *(scale mean scores)* | **Total**  **N=688** |
| --- | --- |
| Ability to self-roster | 6.2 |
| Ability to swap shifts if required | 6.1 |
| Equity for all staff | 5.5 |
| Even spread of skill mix across all shifts | 5.2 |
| Ability to negotiate | 5.0 |
| Adequate rest breaks between shifts | 4.9 |
| Consistency of roster | 4.8 |
| Minimal short-changes between shifts | 3.9 |
| Afternoon shift after days off and morning shift before days off | 3.6 |
