# Supplementary material for: Developing nurse and midwife centred rostering principles using co-design: a mixed-methods study
Source: BMC Nurs. 2024 Dec 20;23:938. doi: 10.1186/s12912-024-02522-7 (PMC11660556; doi:10.1186/s12912-024-02522-7)
Supplement: Supplementary file 2 — Supplementary Material 2 [file 12912_2024_2522_MOESM2_ESM.docx]

**Supplementary Material 2: Unit and roster manager survey data**

**Component 1: Unit and Roster Manager Survey data**

**Table 1: Respondents’ sociodemographic characteristics**

| **Characteristic (n, %)** | **Unit managers**  **(N=14)** | **Roster managers**  **(N=13)** |
| --- | --- | --- |
| **Age** |  |  |
| 26-30 | 0 (0) | 1 (7.7) |
| 31-35 | 2 (14.3) | 3 (23.1) |
| 36-40 | 1 (7.1) | 2 (15.4) |
| 41-45 | 4 (28.6) | 0 (0) |
| 46-50 | 2 (14.3) | 2 (15.4) |
| 51-55 | 4 (28.6) | 5 (38.5) |
| **Years worked as a nurse** |  |  |
| 6-10 years | 0 (0) | 3 (23.1) |
| 10-20 years | 7 (50.0) | 4 (30.8) |
| More than 20 years | 7 (50.0) | 6 (46.2) |
| **Years worked as a midwife** |  |  |
| 10-20 years | 3 (21.4) | 1 (7.7) |
| More than 20 years | 2 (14.3) | 1 (7.7) |
| Not applicable - I have only worked as a nurse | 9 (64.3) | 11 (84.6) |
| **Years been a unit manager** |  |  |
| Less than 1 year | 2 (14.3) |  |
| 1-2 years | 4 (28.6) |  |
| 3-5 years | 2 (14.3) |  |
| 6-10 years | 3 (21.4) |  |
| 10-20 years | 2 (14.3) |  |
| **Years been a unit manager at current health service** |  |  |
| Less than 1 year | 3 (21.4) |  |
| 1-2 years | 4 (28.6) |  |
| 3-5 years | 2 (14.3) |  |
| 6-10 years | 3 (21.4) |  |
| 10-20 years | 2 (14.3) |  |
| **Years been the roster manager in current ward/area** |  |  |
| 0.5 – 1 years |  | 2 (15.4) |
| 1-3 years |  | 4 (30.8) |
| More than 3 years |  | 7 (53.9) |
| **Work status** |  |  |
| Fulltime | 14 (100) |  |
| Part time | 0 |  |
| **% work time non-clinical** |  |  |
| 20% | 1 (7.1) |  |
| 40% | 1 (7.1) |  |
| 80% | 11 (78.6) |  |
| 100% | 1 (7.1) |  |

**Table 2: Roster practices (unit managers)**

| **Item (n, %)** | **Total**  **(N=14)** |
| --- | --- |
| **What participation do you have with the roster on your ward/unit?** |  |
| Complete roster (start to completion) | 2 (14.3) |
| Supervise the writing of roster and contribute as required | 8 (57.1) |
| Only sign off on the roster once completed | 4 (28.6) |
| **How much time do you spend writing/reviewing the roster (in hours)?** (Mean, range) | 4.9 (1─16) |
| **On average, how much time each day do you spend reworking the roster (in hours)?** (Mean, range) | 1. (0─2) |
| **Do you have staff with flexible work arrangements in place?** (‘yes’ responses) | 8 (57.1) |
| **What type of impact does formal flexible work arrangements have when compiling or building the roster?** |  |
| Always a negative impact | 3 (21.4) |
| Slightly negative impact | 6 (42.9) |
| No impact | 1 (7.1) |
| Mostly positive impact | 0 |
| Always positive | 0 |
| Not applicable | 4 (28.6) |
