# Supplementary material for: Developing nurse and midwife centred rostering principles using co-design: a mixed-methods study
Source: BMC Nurs. 2024 Dec 20;23:938. doi: 10.1186/s12912-024-02522-7 (PMC11660556; doi:10.1186/s12912-024-02522-7)
Supplement: Supplementary file 3 — Supplementary Material 3 [file 12912_2024_2522_MOESM3_ESM.docx]

**Supplementary Material 3**

**Component 3: Co-design workshops**

**Table 1: Participant employment and sociodemographic characteristics**

| **Characteristic (n, %)** | **Total**  **N=40*** |
| --- | --- |
| Age (mean, range) | 40.4 (23-61) |
| Role |  |
| Nurse | 32 (80.0%) |
| Midwife | 8 (20.0%) |
| Years practised as a nurse/midwife (mean, range) | 14.2 (0-39) |
| Years worked at current health service (mean, range) | 8.0 (0-23) |

** Although 91 nurses and midwives participated in the co-design workshops, only 40 completed the demographic survey*
