# Supplementary material for: Developing nurse and midwife centred rostering principles using co-design: a mixed-methods study
Source: BMC Nurs. 2024 Dec 20;23:938. doi: 10.1186/s12912-024-02522-7 (PMC11660556; doi:10.1186/s12912-024-02522-7)
Supplement: Supplementary file 5 — Supplementary Material 5 [file 12912_2024_2522_MOESM5_ESM.docx]

**Developing nurse and midwife centred rostering principles**

**Co-design workshop**

**Discussion Guide - Nurses**

*[Thank participants for volunteering; introduce self.]*

*[Go through informed consent process.]*

*[Discuss demographic survey; process for obtaining summary of results]*

*[Reminder that can withdraw at any time, and can choose not to contribute to particular topics.]*

*[Talk about discussion process:*

*Confidentiality;*

*Opportunity for all to ‘speak’;*

*No right or wrong answers]*

“As you know, we have asked nurses and midwives at [name of health service] about their rostering experiences and preferences and based on what they told us we have developed some draft guidelines.

We hope to use the findings of this workshop to refine the guidelines and determine whether further changes are required before they can be used by [name of health service]. We hope the revised guidelines will assist nurses and midwives at [name of health service] to have access more easily to the shifts they prefer and work at times which enable them to manage their personal, family responsibilities, and other competing demands and feel more in control about the times they work and also provide high quality patient care etc.”

We would like to seek your thoughts and preferences on the following roster principles and suggestions which are based on the findings from the surveys and focus groups:

- Adjust the start time of the afternoon shift from 1300H-2130H to 1330H-2200H
- Attempt to align the rostering expectations of managers and staff (i.e. manage staff preferences while ensuring an adequate ward skill mix)
- Allow more flexible start and finish times/shift length
- Create a fatigue management planner
- Create a formal redeployment planner
- Create a night shift planner +/- permanent night shift
- Create education and competency packs relating to rostering
- Explore self-rostering
- Implement longer shift blocks but have less frequent night shift blocks
- Group days off together
- Limit (or totally remove) late earlies (ie day shift after an evening shift) and short changes (ie day shift after a sleeping day off post night shift)
- Prevent last-minute redeployment
- Revise the [interim rostering guidelines](https://westerly.wh.org.au/nursing-midwifery/wp-content/uploads/2022/07/Nursing-Midwifery-Interim-Rostering-Guidelines-2022.pdf) to align with staff flexibility

**What do you think of these suggestions/roster principles overall?**

**Do you think these suggestions/principles need any changes?**

*PROMPTS (after any general discussion)*

Which suggestions/principles? Why/Why not?

Does it cover everything? Should anything be included? Should anything be excluded?

**Do you think the revised suggestions/principles will help nurses and midwives to get the rosters they prefer?**

Why/Why not?

Do you think the guidelines will encourage them/make it easier for nurses and midwives to talk to their NUMs/MUMs etc about their roster needs and preferences?

Do the suggestions/principles provide the flexibility needed to manage your other commitments? Eg childcare, other caring responsibilities, education commitments, other paid work etc? Work/life balance etc

**What do you think are the best ways for nurses and midwives to access the principles?**

Eg WH intranet? NUM/MUM?

Other sugestions?

**Is there anything else you would like to say about the roster suggestions/principles?**

*Thank for participation.*

*We will send results if they leave address: email preferred.*

*Request preferred contact details for gift voucher – email to CI.*

*Remind about demographic survey*
