# Supplementary material for: Developing nurse and midwife centred rostering principles using co-design: a mixed-methods study
Source: BMC Nurs. 2024 Dec 20;23:938. doi: 10.1186/s12912-024-02522-7 (PMC11660556; doi:10.1186/s12912-024-02522-7)
Supplement: Supplementary file 6 — Supplementary Material 6 [file 12912_2024_2522_MOESM6_ESM.docx]

**Developing nurse and midwife centred rostering principles**

**Demographic survey (focus group/co-design workshop participants)**

*Thank you for participating in a focus group or co-design workshop for our project, Developing nurse and midwife centred rostering principles. This information will be used to provide a summary of the participants’ characteristics and only summary data from all the participants and nothing that identifies individuals or their contributions will ever be used or published.*

1. What was your age at your last birthday?

___________________________________________________________________________

1. In which country were you born?

___________________________________________________________________________

1. What is your current position?
   1. RN
   2. EN
   3. Midwife
   4. Nurse & midwife
   5. Nurse Practitioner
   6. Other (*please specify*) ___________________________________________________
2. How many years have you practised as a nurse/midwife?

________________________________________________________________________

1. How many years have you been employed at [name of health service]?

___________________________________________________________________________

1. Which site do you work at?

­­­­__________________________________________________

1. Where do you work? (specific clinical area)
   1. Ward
   2. Theatre
   3. Emergency Department
   4. Maternity
   5. Other (*please specify*)___________________________________________________
