# Supplementary material for: Developing nurse and midwife centred rostering principles using co-design: a mixed-methods study
Source: BMC Nurs. 2024 Dec 20;23:938. doi: 10.1186/s12912-024-02522-7 (PMC11660556; doi:10.1186/s12912-024-02522-7)
Supplement: Supplementary file 7 — Supplementary Material 7 [file 12912_2024_2522_MOESM7_ESM.pdf]

Nursing and Midwifery Roster Survey

Safer Care Victoria (SCV), together with the Department of Health (DH), the Australian Nursing and Midwifery Federation (ANMF Victorian Branch), and three Victorian health services have commenced an employee-centred rostering project. The project aims to address flexibility by creating a set of guidelines that will support more employee-centred rostering.

We are committed to working with you, our front-line nurses, and midwives to develop a set of contemporary employee-centred roster guidelines that best meet your needs.

About the roster project
The project will identify nurses' and midwives' experiences in the changing environment, including your need for flexibility regarding work arrangements, shift allocation, night duty rostering and the importance of ongoing professional development.

The project will involve:

A detailed preliminary online survey Multiple co-designed focus groups and workshops to develop the guidelines A second survey to ascertain if the developed guidelines are suitable and acceptable Follow-up surveys at six- and twelve months following the introduction of the principles and Guidelines to determine how well they are working for nurses, midwives, and health services This survey
We are asking you to complete this survey as you are currently working in a ward/unit at Western Health which has been specifically selected to be involved in this important project. We aim to use this data to drive improvement in rostering guidelines for the nursing and midwifery workforce across Victoria.

We would be most grateful if you could complete the following survey, answering the questions based on your current employment situation. When answering these questions, think about your experience with the rostering process in the last 12 months. The information you provide will be de-identified and, therefore, confidential, so we welcome your honesty in completing this survey.

How will your information be used?
We will ask you for demographic information and we recognise that dependent on where you work this may be minimally identifiable. To ensure the information you provide is protected and you can freely contribute to this project:

No raw information will be viewed by or shared amongst the parties participating in this project Your information will be grouped together with all who responded and only be presented as a whole Project partners will not have access to the surveys. Only the project managers will access survey responses Information will be stored on servers of the health service in which you work
Thank you

#	I have read the plain language statement and understand what is required of me. I understand that proceeding with this questionnaire is considered consent to participate in this project.	◯ Yes<br>◯ No

Demographic Details

1/12 0% Complete

What is your role at your current health service?	◯ ANUM / AMUM<br>◯ GNP / GMP<br>◯ RN / RM / CNS / CMS / EN<br>◯ Other
Please specify:	______________________________
Do you work?	◯ Full Time<br>◯ Part Time
How many hours do you work (per fortnight)?	______________________________
What age range are you in?	◯ < 21<br>◯ 21-25<br>◯ 26-30<br>◯ 31-35<br>◯ 36-40<br>◯ 41-45<br>◯ 46-50<br>◯ 51-55<br>◯ 56-60<br>◯ > 60
How many years have you worked as a nurse?	◯ Less than 1 year<br>◯ 1-2 years<br>◯ 3-5 years<br>◯ 6-10 years<br>◯ 11-20 years<br>◯ More than 20 years<br>◯ Not applicable - I have only worked as a midwife
How many years have you worked as a midwife?	◯ Less than 1 year<br>◯ 1-2 years<br>◯ 3-5 years<br>◯ 6-10 years<br>◯ 11-20 years<br>◯ More than 20 years<br>◯ Not applicable - I have only worked as a nurse
How many years have you worked in your current health service?	◯ Less than 2 years<br>◯ 3-5 years<br>◯ 6-10 years<br>◯ 11-20 years<br>◯ More than 20 years

Roster Survey

2/12 10% Complete.

Do you work a rotating roster across a seven-day week?<br><br>A rotating roster is the ability to be scheduled and rotated through all shifts, across all days of the week	◯ Yes<br>◯ No
Is this due to the role you have?	◯ Yes<br>◯ No
Please elaborate:	__________________________________
What days do you work?	◯ Weekdays only<br>◯ Weekdays and weekends<br>◯ Weekends only
What shifts do you work?<br><br>(tick multiple boxes as relevant)	☐ Days<br>☐ Afternoons<br>☐ Nights<br>☐ Business Hours (e.g., 9-5)
Do you have informal flexible work arrangements in place?<br><br>Informal flexible arrangements are where you can change your work times to accommodate an unexpected personal commitment (e.g., medical appointment)	◯ Yes<br>◯ No<br>◯ I don't know
Do you have a formal flexible work arrangement in place?<br><br>A formal flexible work arrangements is where you have pre -determined arrangements agreed with your manager (e.g., flexi time, compressed work week, job sharing, etc)	◯ Yes<br>◯ No<br>◯ I don't know
Please elaborate:	__________________________________
Do you have responsibilities or commitments that impacts your roster availability?<br><br>(e.g., childcare, care for others, training, volunteering, etc)	◯ Yes<br>◯ No
What type of responsibilities/commitments do you have?<br><br>(tick as many boxes as relevant)	☐ Childcare<br>☐ Caring for others<br>☐ Educational commitments<br>☐ Own health care<br>☐ Volunteering<br>☐ Work elsewhere<br>☐ Other

Please specify: __________________________________

Does your ward/unit have rostering principles/guidelines for staff?

- ◯ Yes
- ◯ No
- ◯ Unsure

Are the rostering principles easily available for all staff to access?

- ◯ Yes
- ◯ No
- ◯ Unsure

Roster Requests

3/12 24% Complete.

Do you have a rostering request system in place?	◯ Yes<br>◯ No<br>◯ Not sure
How many roster requests do you submit on an average in a fortnight?	◯ All shifts<br>◯ 0-1 shifts<br>◯ 2-5 shifts<br>◯ >5 shifts
When you have submitted a roster request over the last 6 months has your request been supported?	◯ Never<br>◯ Rarely<br>◯ Occasionally<br>◯ Usually<br>◯ Always
How satisfied are you with your roster?	Not Satisfied ... Satisfied<br>*(Place a mark on the scale above)*
How many times are you contacted to pick up additional shifts in an average week?	◯ 0<br>◯ 1-2<br>◯ 3-4<br>◯ 5-6<br>◯ >6
How are you contacted?<br><br>(select all that applies)	☐ Phone call<br>☐ SMS<br>☐ WhatsApp<br>☐ Social Media<br>☐ Other
Please specify:	______________________________

Shift Dynamics

4/12 32% Complete.

How many rostered PM shifts followed by an AM shift (without days off in-between) would you work in a fortnight?	◯ None<br>◯ 1-2<br>◯ 3-4<br>◯ 5-6
Would you prefer to be rostered to the same shift for a block of shifts with only one short-change?<br><br>(e.g., 3 morning shifts and 1 evening shift or 3 evening shifts and 1 morning shift)	◯ Yes<br>◯ No<br>◯ No preference
Are your days off rostered together?	◯ Never<br>◯ Sometimes<br>◯ Always
Over the last 6 months how often do you have split days off?<br><br>(split days off is when you have your days off separately in the same week)	◯ Never<br>◯ Sometimes<br>◯ Always
Would you prefer to have your rostered days off together?	◯ Yes<br>◯ No<br>◯ No preference<br>◯ Prefer split days off

Shift Swaps

5/12 37% Complete.

When needing to swap a shift, is there an agreed process in your ward/unit?	◯ Yes<br>◯ No<br>◯ Unsure
How easy is it for you to swap shifts?	Very Difficult ... Very easy<br>*(Place a mark on the scale above)*
Over the last 6 months how often are you successful in swapping a shift?<br><br>(what estimated percentage of time)	Never ... 50% of the time ... All the time<br>*(Place a mark on the scale above)*
On average, how often are you approached to swap a shift in a fortnight?	◯ 0<br>◯ 1-2<br>◯ 3-4<br>◯ 5-6<br>◯ >6
Are you predominantly approached by the manager or colleagues to swap shifts?	◯ Manager<br>◯ Colleague<br>◯ Both
To maintain work/life commitments, how far in advance would you like your roster published?	◯ 2 weeks<br>◯ 4 weeks<br>◯ 6 weeks<br>◯ 8 weeks<br>◯ 10 weeks or more

Please RANK in order from 1 to 9 how important the following factors are in your roster

(1 is the least important and 9 is the most important, each number can only be used once)

	1	2	3	4	5	6	7	8	9
Ability to self-roster	○	○	○	○	○	○	○	○	○
Ability to swap shifts if required	○	○	○	○	○	○	○	○	○
Equity for all staff on roster	○	○	○	○	○	○	○	○	○
Ability to negotiate	○	○	○	○	○	○	○	○	○
Consistency of roster	○	○	○	○	○	○	○	○	○
Even spread of skill mix across all shifts	○	○	○	○	○	○	○	○	○
Minimal short-changes between shifts	○	○	○	○	○	○	○	○	○
Adequate rest breaks between shifts	○	○	○	○	○	○	○	○	○
Afternoon shift after days off and morning shift before days off	○	○	○	○	○	○	○	○	○

Night shifts

6/12 54% Complete.

If you rotate onto night shifts, on average over the last 6 months how many weeks do you spend on night shifts for each rotation?	◯ Less than 1 week<br>◯ 1 week<br>◯ 2 weeks<br>◯ 3 weeks<br>◯ 4 weeks<br>◯ 5 weeks or more<br>◯ Not applicable
How many night duty shifts does it take for your body to acclimatise to working night duty?	◯ 1-2<br>◯ 3-4<br>◯ Week<br>◯ Not applicable<br>◯ I never acclimatise
Would being able to choose when you are rostered to do night shifts improve your satisfaction with your roster?	◯ Yes<br>◯ No<br>◯ Not applicable
How often would you like to do night duty in a 12-month period?	◯ Never<br>◯ 2 weeks a year<br>◯ 4 weeks a year<br>◯ 8 weeks a year<br>◯ Greater than 8 weeks
Would you like to complete night shift in a block or mixed in with day and afternoon shifts?	◯ One block<br>◯ Mixed with other shift times
Would the option of permanent night duty suit you?	◯ Yes<br>◯ No

Rostering

7/12 61% Complete.

Would having more flexible rostering guidelines improve your work schedule?	◯ Yes<br>◯ No<br>◯ Unsure
Which of the following would increase your satisfaction with your roster?<br><br>(please select all that apply)	☐ Full self-roster with underlying rules<br>☐ Request roster system with clear rules<br>☐ Choice of set rosters<br>☐ Other
Please specify:	__________________________________
Over the last 6 months, have you had to take personal leave as your roster has resulted in fatigue?<br><br>(please note that this survey is anonymous)	◯ Yes<br>◯ No
Over the last 6 months, have you had to take personal leave as your roster requests have been unable to be met?<br><br>(please note that this survey is anonymous)	◯ Yes<br>◯ No

Skill mix

8/12 66% Complete.

In your opinion, how often is there a less than ideal skill mix of staff on shifts in your ward/unit?	◯ Rarely<br>◯ Often<br>◯ Very often
When there is an uneven skill mix on shifts, how much does this effect your ability to perform your duties and deliver care?	◯ No effect<br>◯ Minor effect<br>◯ Neutral<br>◯ Moderate effect<br>◯ Major effect<br>◯ Not applicable

Roster Flexibility

9/12 69% Complete.

Does your ward/unit use a supplementary roster?<br><br>(i.e., where you can volunteer your availability to work additional shifts over your contracted hours)	◯ Yes<br>◯ No<br>◯ Not sure
Are you asked to work additional shifts when you haven't stated that you can work extra shifts?	◯ Yes<br>◯ No<br>◯ Not applicable
Have you ever needed to request temporary flexibility in your roster due to a personal commitment, and if yes, how often?	◯ At least weekly<br>◯ At least once a month<br>◯ At least every 6 months<br>◯ Very infrequently<br>◯ Never
How would you rate the flexibility within your ward/unit if you have a legitimate need to do something different with your roster on a particular day?<br><br>(e.g., start a bit late, leave a bit early)<br><br>A legitimate reason may include medical appointments, parent-teacher interviews, Ramadan etc.	◯ Very flexible<br>◯ Flexible depending on circumstances<br>◯ Somewhat flexible<br>◯ Not flexible at all<br>◯ Have not needed flexibility

Short shifts

10/12 73% Complete.

Have you experienced working short shifts?<br><br>(shifts that are shorter than 8 hours for an AM/PM or 10 hours for night duty)	◯ Yes<br>◯ No
Would you prefer the option of short shifts as a regular feature in your roster?	◯ Yes<br>◯ No
Would this suit your work-life balance if short shifts were available in your area?	◯ Yes<br>◯ No<br>◯ Doesn't make a difference
If short shifts were available, how many would you request per fortnight?	◯ 0<br>◯ 1<br>◯ 2<br>◯ 3<br>◯ 4<br>◯ 5<br>◯ All my roster
What would your preferred start time be for a short shift?	________________________
What is your preferred short shift length during the day (in hours)?	________________________
Would you prefer the regular 8-8-10 shifts to be..	◯ Longer<br>◯ Shorter<br>◯ Stay the same
How long a shift would you prefer (in hours)?	________________________
Would you prefer to alter the length of the current night duty shift?	◯ Yes<br>◯ No
What is your preferred night shift length (in hours)?	________________________

Clinical area

11/12 84% Complete.

Which clinical area are you currently employed?<br><br>(within the health service)	◯ Medical<br>◯ Surgical<br>◯ Subacute nursing<br>◯ Emergency<br>◯ Midwifery<br>◯ Coronary care services
Please enter what area:	__________________________________
Do you regularly rotate through more than one area of speciality?<br><br>(i.e., Antenatal to Birthing or similar)	◯ Yes<br>◯ No
How long are your rotations?	◯ 1-2 shifts a week<br>◯ 3-4 shifts a week<br>◯ 1-3 week block<br>◯ Greater than 4 weeks
Does the length of your rotations support maintenance of your clinical confidence in the area?	◯ Yes<br>◯ No
Does the length of your rotation provide you with adequate growth of your knowledge and skill set in a particular area?	◯ Yes<br>◯ No
What is your preferred duration for a rotation?	◯ 1 shift<br>◯ 3-4 shifts a week<br>◯ 1-3 week block<br>◯ Greater than 4 weeks<br>◯ No preference
Would you prefer to work within one specific area of speciality?	◯ Yes<br>◯ No<br>◯ I don't mind
How often are you redeployed to a different area on the day of your shift?	◯ Nil<br>◯ Once per week<br>◯ Twice per week<br>◯ Three times per week<br>◯ Four times per week<br>◯ Five times per week
Do you feel that redeployment on the day of a shift is managed in a fair manner within your ward/unit?	Totally unfair — Neither — Totally fair<br><br>*(Place a mark on the scale above)*
Please elaborate:	__________________________________

Almost there

12/12 96% Complete.

Reflecting on your previous responses, what are the positive aspects of the rostering process in your current ward/unit?	☐ Flexibiliity<br>☐ Accomodating<br>☐ Fair<br>☐ Ability to request<br>☐ Other
Please elaborate:	______________________________
Have we missed anything? Please let us know if there is anything else you would like to tell us about the rostering process at WH.	______________________________

Thank you for taking the time to complete this survey. We appreciate the time you have taken to assist in the collection of valuable information in rostering processes at your health service.

We are committed to utilising the information gained to contemplate and implement worthwhile improvements for rostering for nurses and midwives.

Everyone who completes a survey can enter the draw for one of ten $100 gift vouchers.<br><br>Do you wish to enter the gift voucher draw?<br><br>(so that your responses to the survey remain anonymous, you will be directed to a separate form to register your interest in the gift voucher draw)	◯ Yes<br>◯ No
