# Supplementary material for: Developing nurse and midwife centred rostering principles using co-design: a mixed-methods study
Source: BMC Nurs. 2024 Dec 20;23:938. doi: 10.1186/s12912-024-02522-7 (PMC11660556; doi:10.1186/s12912-024-02522-7)
Supplement: Supplementary file 8 — Supplementary Material 8 [file 12912_2024_2522_MOESM8_ESM.docx]

**Developing nurse and midwife centred rostering principles**

**Pre-implementation Discussion Groups**

**Discussion Guide**

*[Thank participants for volunteering; introduce self.]*

*[Go through informed consent process.]*

*[Discuss demographic survey; process for obtaining summary of results]*

*[Reminder that can withdraw at any time, and can choose not to contribute to particular topics.]*

*[Talk about discussion process:*

*Confidentiality;*

*Opportunity for all to ‘speak’;*

*No right or wrong answers]*

Overview of study

WH/SCV are keen to support the wellbeing of nurses and midwives are concerned that nursing and midwifery staff are often dissatisfied with their rosters and current rostering guidelines, and this maybe having a negative effect on their job satisfaction, wellbeing and ability to deliver high quality patient/best care etc.

The aim of this project is to explore nurses' and midwives' experiences, perceptions of and satisfaction with the current rostering system; identify nurses' and midwives' rostering preferences; and co-design and evaluate revised roster guidelines

**Overarching themes that may be explored in the focus groups are:**

1. Shift patterns (incl. blocks of shifts, what is time off considered, start/finish times, lengths, finishing on AM and starting on PM)
2. Understanding the high satisfaction % but wanting more flexibility
3. Night duty (incl. permanent ND, should ND have a higher penalty rate across the board, professional development, expectations to come onto days)
4. What are the barriers and challenges (incl. what is driving these, childcare)
5. How far in advance do they want the roster - how often does it change after being published
6. Working arrangements (incl. how many informal agreements exist
7. Supplementary rosters (incl. language, inconsistencies, clear pathways to make yourself available)
8. Rostering bias - what does having a fair roster mean, fairness between part time/full time
9. Solutions

**What do you think of the current roster guidelines at WH overall?**

Impact of shift work on health

**How do the current roster guidelines/shift work impact our health?**

*Sleep - Do you have a work schedule that sometimes overlaps with the time you usually sleep? Does this cause insomnia and/or excessive sleepiness due to reduced amount of sleep?*

*Wellbeing*

Impact of shift work on personal and family responsibilities

**How do the current roster guidelines/shift work affect your ability to manage your paid work and family responsibilities?**

*Eg child care etc*

Impact on nursing/midwifery care

**Do the current roster guidelines/schedule impact your ability to provide high quality care?**

*How? Missed elements of patient care?*

Turnover intentions/job satisfaction

**Have you thought about leaving WH/nursing/midwifery because the current roster guidelines/practices don’t meet your needs?**

**Do the current roster guidelines/scheduling impact your job satisfaction?**

*How?*

Perceptions of fairness and equity

**Do you think the current roster guidelines are fair and equitable?**

*Why?/Why not?*

Rostering preferences/needs

**Do the current roster guidelines meet your needs?**

*Why/Why not?*

**What would increase your satisfaction with your roster?**

*Eg Ability to self-roster? Easier to swap shifts? Minimal short-changes between shifts? Adequate rest breaks between shifts? Fewer blocks of night shift? More flexibility? Different shift start/finish times?*

**Is there anything else you would like to say about rostering for nurses and midwives at WH?**

*Thank for participation.*

*We will send results if they leave address: email preferred.*

*Request preferred contact details for gift voucher – email to CI.*

*Remind about demographic survey*
